# RNA-seq profiling in leaf tissues of two soybean (*Glycine max* [L.] Merr.) cultivars that show contrasting responses to drought stress during early developmental stages

**DOI:** 10.1007/s11032-023-01385-1

**Published:** 2023-05-09

**Authors:** Xuefei Yang, Hakyung Kwon, Moon Young Kim, Suk-Ha Lee

**Affiliations:** 1 Key Laboratory of Herbage & Endemic Crop Biology of Ministry of Education, School of Life Sciences, Inner Mongolia University, Hohhot, 010030 China; 2 Department of Agriculture, Forestry and Bioresources and Research Institute of Agriculture and Life Sciences, Seoul National University, Seoul, 08826 Republic of Korea; 3 Plant Genomics and Breeding Institute, Seoul National University, Seoul, 08826 Republic of Korea; 4 Crop Genomics Lab., Seoul National University, Rm. 4105 Bldg. 200 CALS, 1 Gwanak-ro, Gwanak-gu, Seoul, 08826 Republic of Korea

**Keywords:** Drought tolerance, Early vegetative stage, Lipid-signaling pathway, Phosphatidyl inositol monophosphate 5 kinase, RNA sequencing, Soybean

## Abstract

Drought stress is the major environment constraint on soybean yield, and a variety of pathways underlie drought tolerance mechanisms. Transcriptomic profiling of two soybean cultivars, drought-tolerant SS2-2 and drought-sensitive Taekwang, was performed under normal and drought conditions to identify genes involved in drought tolerance. This revealed large differences in water loss during drought treatment. Genes involved in signaling, lipid metabolism, phosphorylation, and gene regulation were overrepresented among genes that were differentially expressed between cultivars and between treatments in each cultivar. The analysis revealed transcription factors from six families, including WRKYs and NACs, showed significant SS2-2-specific upregulation. Genes involved in stress defense pathways, including MAPK signaling, Ca^2+^ signaling, ROS scavenging, and NBS-LRR, were also identified. Expression of non-specific phospholipases, phospholipase D, and *PHOSPHATIDYL INOSITOL MONOPHOSPHATE 5 KINASE* (*PIP5K*), which act in the lipid-signaling pathway, was greatly increased in SS2-2. The roles of *PIP5K* in drought stress tolerance were confirmed in *Arabidopsis thaliana*. *Arabidopsis* *pip5k* mutants had significantly lower survival rates under drought stress than wild-type plants. This study identified additional elements in the mechanisms used by plants to protect themselves from drought stress and provides valuable information for the development of drought-tolerant soybean cultivars.

**Supplementary Information:**

The online version contains supplementary material available at 10.1007/s11032-023-01385-1.

## Introduction

Soybean (*Glycine max* (L.) Merr.) contains high levels of protein and oil and is one of the most economically important crops worldwide. Soybean growth and yield, however, are severely affected by various abiotic stresses and, in particular, by drought stress, which may reduce productivity by up to 40% (He et al. 2017). Furthermore, episodes of water scarcity are occurring more frequently across the world due to global climate change and limited arable lands (Koncagül et al. 2018). Thus, understanding the mechanisms of drought tolerance in soybean can help to develop cultivars that use water more efficiently and remain high yielding.

Crop plants have evolved a range of regulatory strategies to enhance resistance to drought stress at the morphological, developmental, and physiological levels (Basu et al. 2016; Rellán-Álvarez et al. 2016; Dinneny 2019). This process involves three steps: sensing the environmental stimuli, transmitting the signals, and responding to them. When plants sense water loss from the soil, the roots generate a small peptide called CLE25 (Takahashi et al. 2018) to transmit water-deficiency signals over long distances and affect the abscisic acid (ABA) biosynthesis pathway in leaf tissues. Hyperosmotic stress caused by drought can change the phospholipid tension, which is sensed by Ca^2+^ channels, generating Ca^2+^ spikes (Yuan et al. 2014). These Ca^2+^ signals can then result in downstream transcriptional activation by binding to calmodulin-binding transcription factors (Kim et al. 2013). Drought stress can also induce ROS production (Hipsch et al. 2021), which can serve as secondary messengers to transmit the stress signal, causing ABA accumulation and further stomatal closure. CLE25, Ca^2+^, and ROS can all lead to ABA-mediated stress responses to drought (Song et al. 2016a).

Furthermore, drought stress can induce genome-wide transcriptional reprogramming, with many transcription factors involved in the drought stress response, including ABA-dependent and ABA-independent pathways (Zhang et al. 2022). ABA-dependent genes contain the ABA-responsive cis-element (ABRE) in their promoter regions and are regulated by ABRE-binding factors (ABFs), whereas ABA-independent drought-responsive genes often contain the dehydration-responsive cis-element (DRE) and are regulated by the AP2/ERF (Apetala2 and ethylene-responsive factors) transcription factor family (Yamaguchi-Shinozaki and Shinozaki 2006; Maruyama et al. 2012). Besides, many other transcription factors were also reported to respond to drought stress, such as WRKY, MYB, NF-Ys, and NACs, through an ABA-dependent manner, or some NACs through an ABA-independent manner (Yao et al. 2021; Singh and Laxmi 2015).

High-throughput expression profiling technology has revolutionized our understanding of soybean’s molecular responses to drought stress. Several transcriptomic studies have been conducted on soybean to identify drought-responsive genes across different developmental stages and tissues (Fan et al. 2013; Shin et al. 2015; Song et al. 2016b). Some common drought-responsive genes have been identified across different cultivars under water-deficit treatment, suggesting that these genes are conserved in soybean. Furthermore, some DEGs related to photosynthesis and lipid metabolism were found to be involved in environmental response with no genotypic difference (Shin et al. 2015). The early molecular responses of soybean to stresses were investigated by exposing the seedlings of the soybean line HJ-1 to different stresses for 48 h. The study revealed some genes that showed similar expression patterns across different stress treatments, indicating the presence of crosstalk among stress responses (Fan et al. 2013). Another study identified the transcriptomic changes induced in the primary roots of the model cultivar Williams 82 at the V3 stage under varying levels of water-deficit stress. The identified genes were found to be involved in regulating taproot responses to water-deficit stress (Song et al. 2016b).

While several transcriptomic profiling studies have been conducted to investigate the mechanisms underlying drought resistance in soybean, they have primarily focused on instant dehydration stress treatments or the post-vegetative growth period (Chen et al. 2013; Prince et al. 2015; Shin et al. 2015). However, in field conditions, crops typically experience prolonged and moderate water deficit, which is more representative of actual drought stress (Gupta et al. 2020). Drought stress during the reproductive stage in most soybean genotypes causes irreparable damage due to direct flower abortion and early drop of young pods, ultimately decreasing yield (Farooq et al. 2017). Nevertheless, during the early developmental stages (i.e., before flowering), different soybean genotypes respond differently to drought stress which subsequently affects yield as well. Several soybean genotypes maintain high values of traits such as leaf area and chlorophyll content despite encountering drought stress at the vegetative stage (Yan et al. 2020), and some are able to recover from drought injuries after rewatering, even exhibiting compensation effects on growth (Don et al. 2019). Soybean genotypes displaying drought tolerance during their early developmental stages undergo little damage and show small decreases in yield (Yan et al. 2020). Unveiling the molecular mechanisms underlying drought tolerance during the early vegetative stages will therefore facilitate the development of drought-tolerant soybean cultivars. Furthermore, although previous transcriptomic studies identified gene ontology pathways enriched in sets of genes involved in drought responses, they did not confirm the function of individual genes in enhancing drought tolerance. Transcriptomic resources derived from more extensive biological samples are required to explore the molecular modules mediating drought tolerance in soybean. Such resources will aid the development of high-yielding soybean cultivars tolerant to drought, in particular exposure to a prolonged dry period after seed germination. Therefore, the current study was designed to identify genes responsible for drought tolerance at the early vegetative stage undergoing a prolonged moderate drought. We screened 11 soybean genotypes and identified two cultivars, SS2-2 and Taekwang, that were resistant and sensitive to drought stress, respectively, and showed contrasting wilting phenotypes under prolonged water restriction. By comparing transcriptomic data obtained from the leaves of these two genotypes, we identified a set of differentially expressed genes (DEGs) that enhanced drought tolerance. The biological function of one of these genes, *GmPIP5K*, in enhancing drought tolerance was investigated and confirmed by knockout studies of the homologous gene in *Arabidopsis* (*Arabidopsis thaliana*).

## Materials and methods

### Plant materials and drought treatments

This study initially investigated 11 soybean genotypes (the model cultivar Williams 82, Keunol, SS2-2, Buseok, Iksannamul, Jiyu69, SS0404-T5-76, Danbeak, Jangyeob, Taekwang, and Cheongja 3) to select drought-tolerant and drought-sensitive genotypes for transcriptomic analysis. All these genotypes, except Williams 82, have served as parental lines in crosses that produced recombinant inbred line (RIL) populations (Sun et al. 2013). Each soybean plant was grown in a 1.5-L pot containing the same quantity of sterilized commercial peat soil (around 600 g in dry weight) to prevent nodulation. The same volume of water was provided every day with a volume of 20 mL, and the locations of the pots were rotated to avoid differential drying of the soil. When the third trifoliate leaves were completely open (V3 stage), watering was discontinued as the drought treatment. For the primary screen, the relative water content (RWC) of the third trifoliate leaf was recorded every 4 days until the plant withered. To calculate RWC, leaves were collected from each genotype, and the fresh weights were recorded. The leaves were soaked completely in water for 4–6 h to obtain the saturated weight. To obtain the dry weight, the leaves were incubated at 65°C for 2–3 days until the tissues were completely dry. RWC is an expression of the water content as a percentage relative to the water content at full turgor: (fresh weight – dry weight)/ (saturated weight – dry weight) × 100.

A second screen was performed using five genotypes (Jangyeob, Taekwang, Cheongja 3, Iksannamul, and SS2-2). RWC was measured in each of the first (V1), second (V2), and third (V3) trifoliate leaves 7 and 14 days after water restriction (DAWR). After the final selection of the drought-tolerant and drought-sensitive genotypes, we again evaluated the drought sensitivity of the genotypes by measuring RWC in the third (V3) and fourth (V4) trifoliate leaves every 4 days in the absence of water.

To sample leaf tissues for RNA-seq analysis, the same drought treatment was applied to the selected genotypes, SS2-2 and Taekwang. After an 8-day drought treatment, trifoliate leaves were collected from both genotypes using three biological replicates. The plants reached the V4 stage after the treatment and, as a control, V3 leaves from plants grown under normal conditions were used.

### RNA isolation and RNA sequencing

Total RNA was extracted from leaf samples using GeneAll Ribospin™ Plant Kit (Cat. 307-150; GeneAll, Seoul, Korea) according to the manufacturer’s instructions. After a check for quality control, cDNA libraries were constructed using a TruSeq Stranded mRNA LT Sample Prep Kit. The sequencing libraries were prepared by random fragmentation of the cDNA sample, followed by 5′ and 3′ adapter ligation. Next, adapter-ligated fragments were amplified by PCR and gel purified. The cDNA libraries were sequenced on an Illumina HiSeq400 platform with a read length of 100 bp and a paired-end read type.

### Read alignment and RNA-seq analysis

Raw reads were indexed using Bowtie2 (http://bowtie-bio.sourceforge.net/index.shtml) and mapped to the soybean reference genome (Gmax_Wm82_a2_v1) using TopHat (http://ccb.jhu.edu/software/tophat/index.shtml). The Cufflinks program was used to assemble gene transcripts and to normalize transcript abundance in terms of fragments per kilobase pairs of fragments per million mapped reads (FPKM). The count number was calculated using the script HTseq-count in the HTseq package. The EdgeR (https://bioconductor.org/packages/release/bioc/html/edgeR.html) was used to test statistically significant differences in transcriptional expression between cultivars and under the two treatment conditions, namely, control and drought. Differentially expressed genes (DEGs) were identified using a criterion of a twofold expression change (*P* value < 0.01) (Fig. S1).

### Functional classification and enrichment of DEGs by GoMapman, BiNGO, and KEGG

The protein functions of DEGs were categorized using the annotation of GoMapman (http://www.gomapman.org/ontology). The gene ontology (GO) enrichment analysis of DEGs was assessed using Biology Networks Gene Ontology Tools (BiNGO) (http://www.psb.ugent.be/cbd/papers/BiNGO/Home.html) to determine significantly overrepresented terms. Significantly overrepresented GO categories were visualized in Cytoscape (http://www.cytoscape.org). The metabolism pathways in which DEGs were likely to act were determined using the Kyoto Encyclopedia of Genes and Genomes (KEGG; https://www.genome.jp/kegg/). The scatterplots of enriched KEGG pathways of DEGs were assessed using the ClusterProfiler package in R software (Yu et al. 2012).

### Quantitative real-time PCR (qRT-PCR) analysis of DEGs

qRT-PCR analyses were used to confirm the expression levels of selected DEGs identified in the RNA-seq data. Total RNA was extracted using GeneAll Ribospin™ Plant Kit (Cat. 307-150; GeneAll, Seoul, Korea). Approximately 1 μg of total RNA was reverse transcribed using an iScript™ cDNA Synthesis Kit (Cat. 1708891; Bio-Rad, CA, USA). qRT-PCR was performed using iQ™ SYBR® Green Supermix (Cat. 170-8880AP; Bio-Rad) in a LightCycler 480 Real-Time PCR system (Roche Diagnostics, Laval, QC, Canada). The soybean housekeeping gene *ACTIN11* was used as a reference gene (Jian et al. 2008). All primers were designed using primer3 (http://bioinfo.ut.ee/primer3-0.4.0/). The primer sequences used in this study are listed in Table S1.

Each sample consisted of three biological replicates, each with two technical replicates. The statistical significance of differences between groups was determined using Student’s *t-*test.

### Functional validation of DEGs using *Arabidopsis* knockout mutants

Seeds of *Arabidopsis* T-DNA insertion lines in which the target genes had been knocked out were obtained from the Arabidopsis Biological Resource Center (ABRC, http://abrc.osu.edu/) at Ohio State University, USA. To check the seed germination rates of *Arabidopsis* lines under osmotic and salt stress, 100 seeds of the wild-type line Col-0 and each mutant line were surface-sterilized and sown on 0.5× MS medium containing either 300 mM mannitol or 150 mM NaCl. The number of germinating seeds was counted at 7 days after sowing. To determine the survival rates of *Arabidopsis* seedlings under drought stress treatment, Col-0 and mutant seeds were germinated on the soil. Individual 1-week-old seedlings were transplanted into pots containing an equal quantity of soil. The pots were soaked in water for 10 min and placed in a growth chamber without further watering. Ten days later, the pots were rewatered to check the survival rate on the next day. Statistical significant differences between groups were determined by Student’s *t*-test using data from three independent experiments.

## Results

### SS2-2 and Taekwang show opposite responses to drought stress

**Fig. 1:**
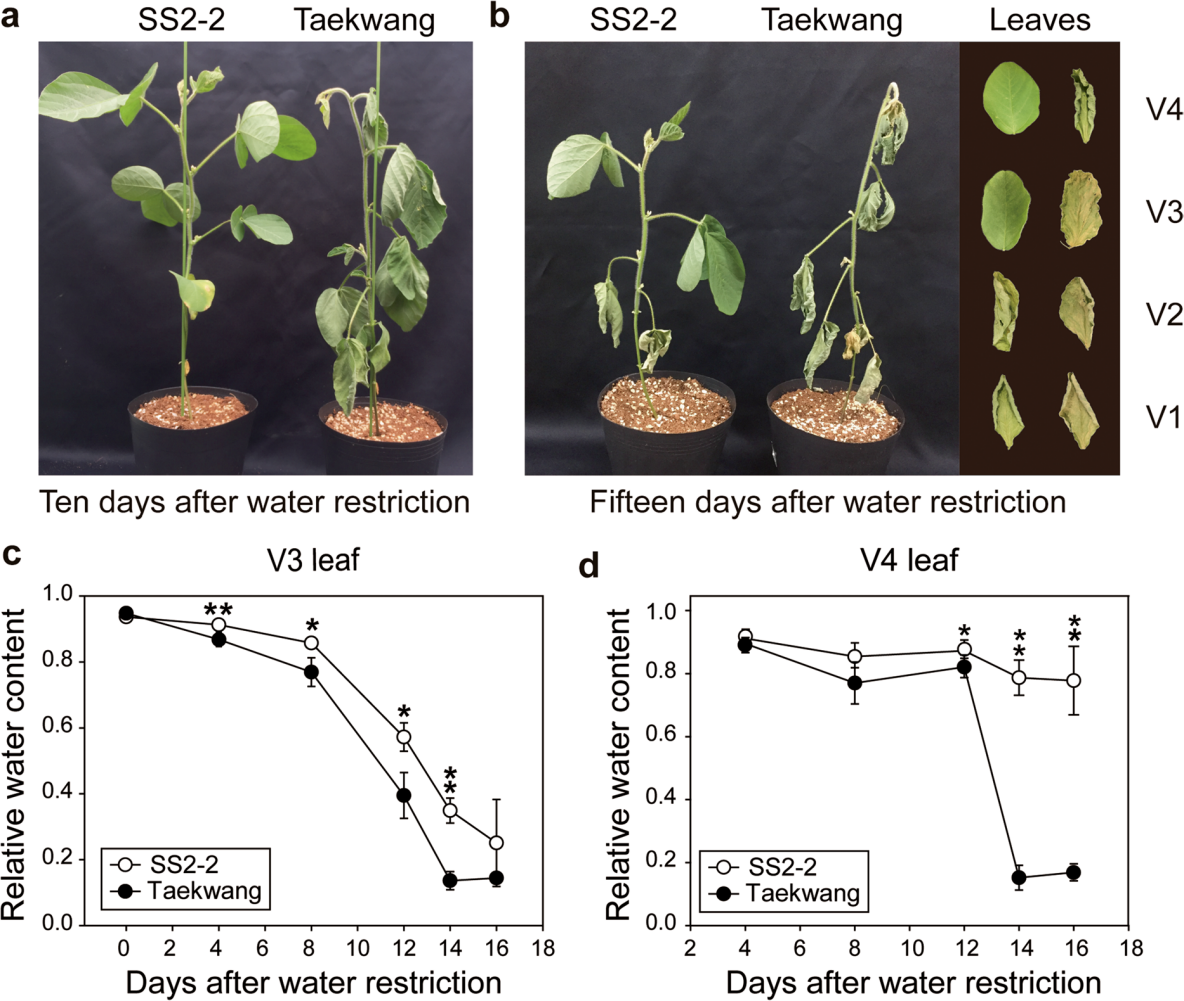
Responses of SS2-2 and Taekwang to drought stress**. a** Representative SS2-2 and Taekwang plants 10 days after water restriction (DAWR). **b** Representative SS2-2 and Taekwang plants 15 DAWR. Note that leaves V1–4 showed different rolling patterns due to their different relative water content (right-hand panel). **c, d** Relative water content (RWC) of (**c**) V3 leaves and (**d**) V4 leaves measured every 4 days after the discontinuation of water

Measurements of RWC in V3 leaves of 11 different soybean genotypes exposed to drought stress revealed substantial variation in drought sensitivity between the genetic backgrounds (Fig. S2). At 4 DAWR, no significant differences in RWC were observed. The RWC of Taekwang, however, had dropped considerably by 8 DAWR, while SS2-2 maintained the highest RWC until 12 DAWR. Based on these results, five genotypes (SS2-2, Iksannamul, Cheongja 3, Jangyeob, and Taekwang) were selected for inclusion in the second screen (Fig. S3). By 7 DAWR, Jangyeob and Taekwang showed significant decreases in RWC in all leaves compared to SS2-2, Iksannamul, and Cheongja 3 (Fig. S3a). At 14 DAWR, RWC was higher in the V3 leaves of SS2-2 and Iksannamul than in those of Cheongja 3; moreover, leaves of the former genotypes retained their normal shape with little wrinkling (Fig. S3b). As the V3 leaves of SS2-2 and Taekwang showed the highest and lowest RWC, respectively, in the second screen, they were subjected to the final drought experiment. The RWC of V3 leaves from SS2-2 and Taekwang differed significantly from 4 DAWR and sharply decreased between 8 and 14 DAWR in both genotypes (Fig. 1a). SS2-2 also maintained a higher RWC in its V4 leaves than Taekwang (Fig. 1b). After 10 days without watering, SS2-2 still showed vigorous growth, but the leaves of Taekwang were shriveled, withering completely by 15 DAWR (Fig. 1c, d). These drought tests revealed that these two genotypes displayed contrasting tolerance to a high level of drought stress. SS2-2 and Taekwang were therefore designated “drought-tolerant” and “drought-sensitive” genotypes, respectively.

### RNA-seq analysis identified DEGs under normal and drought conditions

To investigate the mechanism underlying the contrasting levels of drought tolerance in SS2-2 and Taekwang, we performed a comparative transcriptomic study. V3 leaves were collected from both genotypes under control conditions and at 8 DAWR, when their RWC began to decrease dramatically (Fig. 1a). An average of 66 million reads per sample was mapped against the soybean reference sequence with an average mapping rate of 81.26% (Table S2).

**Fig. 2:**
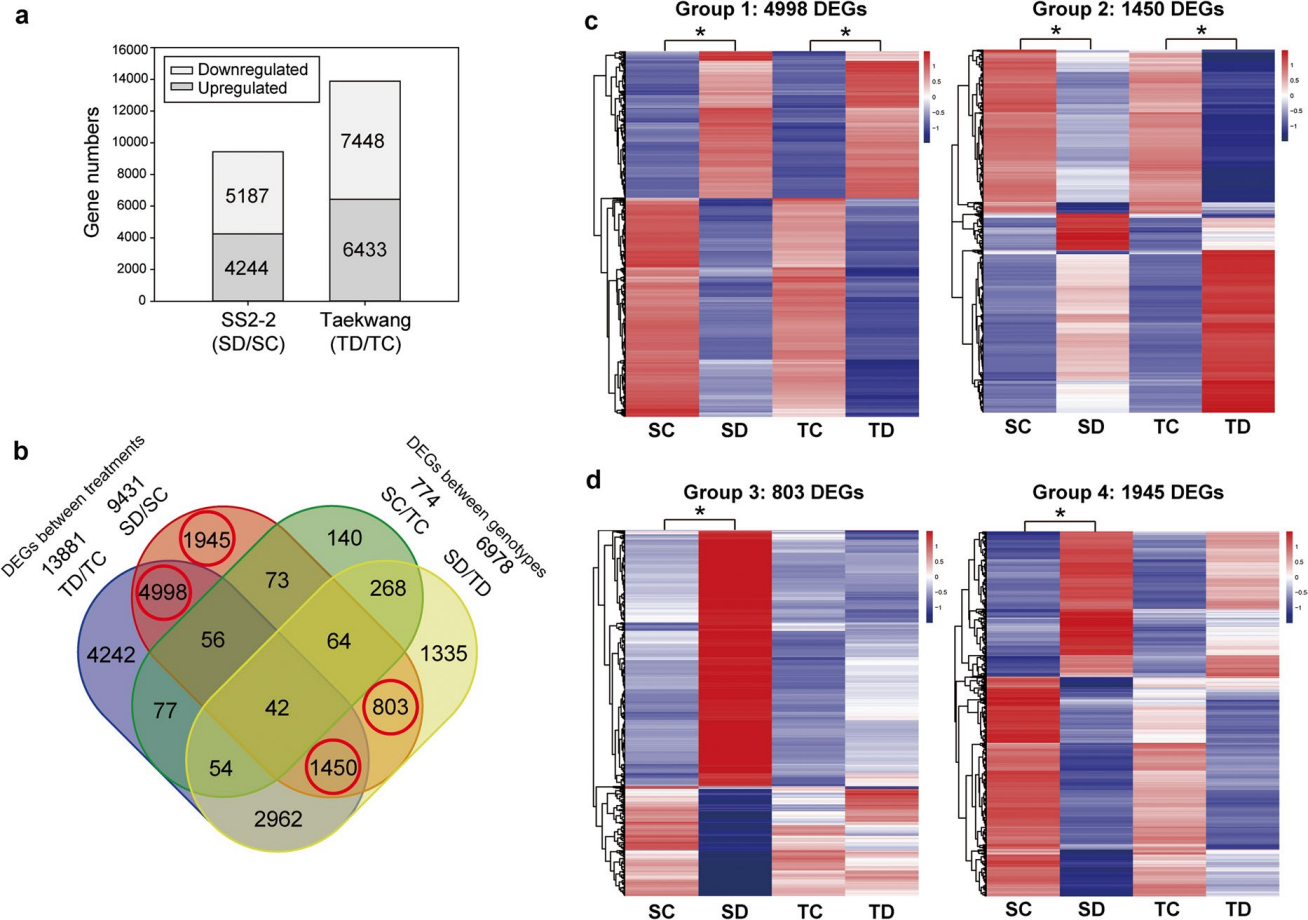
An RNA-seq analysis identified differentially expressed genes (DEGs) between SS2-2 and Taekwang. **a** The numbers of upregulated and downregulated genes in SS2-2 (SD/SC) and Taekwang (TD/TC) under drought treatment. **b** Venn diagram showing the overlaps between DEGs that are compared between genotypes or treatments (SC/SD, TC/TD, SC/TC, and SD/TD). **c, d** Heatmap showing the expression level of four groups of DEGs that were differentially expressed in SS2-2 between control and drought stress conditions but were not differentially expressed between genotypes under control conditions (marked as red circles in the Venn diagram). **c** Genes that were differentially expressed in both SS2-2 and Taekwang. **d** Genes that were differentially expressed in SS2-2 but not in Taekwang between control and drought stress conditions. *Significant at *P* < 0.05

To identify differentially expressed genes (DEGs) responsible for drought tolerance, we conducted a transcriptomic comparison between different treatments and genotypes. Specifically, we compared the transcript abundance of SS2-2 control (SC) and drought (SD) treatments, as well as Taekwang control (TC) and drought (TD) treatments. We found 9431 DEGs in SS2-2 (SD/SC) and 13,881 DEGs in Taekwang (TD/TC). Of the 9431 DEGs in SS2-2, 4244 were upregulated and 5187 were downregulated in drought treatment compared to control (Fig. 2a). In Taekwang, 6433 genes of the 13,881 DEGs were upregulated and 7448 genes downregulated during drought stress (Fig. 2a). To further analyze the DEGs, we categorized the DEGs into different groups based on their genotype-related and drought-related genes using a Venn diagram (Fig. 2b). Between the two genotypes, we identified 774 genes that showed differences in expression in control conditions (SC/TC) and 6978 genes whose expression differed under drought stress (SD/TD) between the two genotypes.

As SS2-2 was found to be a drought-tolerant genotype, we selected four groups of SC/SD DEGs (circled in red in Fig. 2b) to examine in detail. We displayed their expression level using a heatmap (Fig. 2c, d). Two groups containing 4998 (group 1) and 1450 genes (group 2) were significantly induced by drought stress in both SS2-2 and Taekwang (Fig. 2c), and were considered common DEGs showing similar responses to drought stress in both genotypes. The other two groups, containing 803 (group 3) and 1945 (group 4) genes, respectively (2748 genes in total), were differentially expressed in SS2-2 in response to drought, but showed no changes in expression in Taekwang. Therefore, we considered them as SS2-2-specific DEGs (Fig. 2d, Table S3). These 2748 SS2-2-specific DEGs consisting of 1336 genes upregulated and 1412 DEGs downregulated genes under drought stress were further studied.

We used GoMapman to perform a functional classification of DEGs varying between control and drought treatment. This showed that “protein synthesis” and “RNA processing” were the most highly enriched categories in both SS2-2 and Taekwang (Fig. S4). The term “signaling,” which is important in stress signal transduction, was ranked third in SS2-2 (11.7% of DEGs) and fourth in Taekwang (8.6% of DEGs). Genes related to “stress” made up 7.3% of SS2-2 DEGs, which was higher than the 5.5% observed in Taekwang. In addition, the protein categories “transport,” “hormone metabolism,” and “lipid metabolism” all showed higher enrichment in SS2-2 than in Taekwang (6.8% vs 6.6%, 3.7% vs 3.0%, and 3.0% vs 3.7% in SS2-2 vs Taekwang, respectively).

### Functional classification of SS2-2-specific DEGs by BiNGO and KEGG

**Fig. 3:**
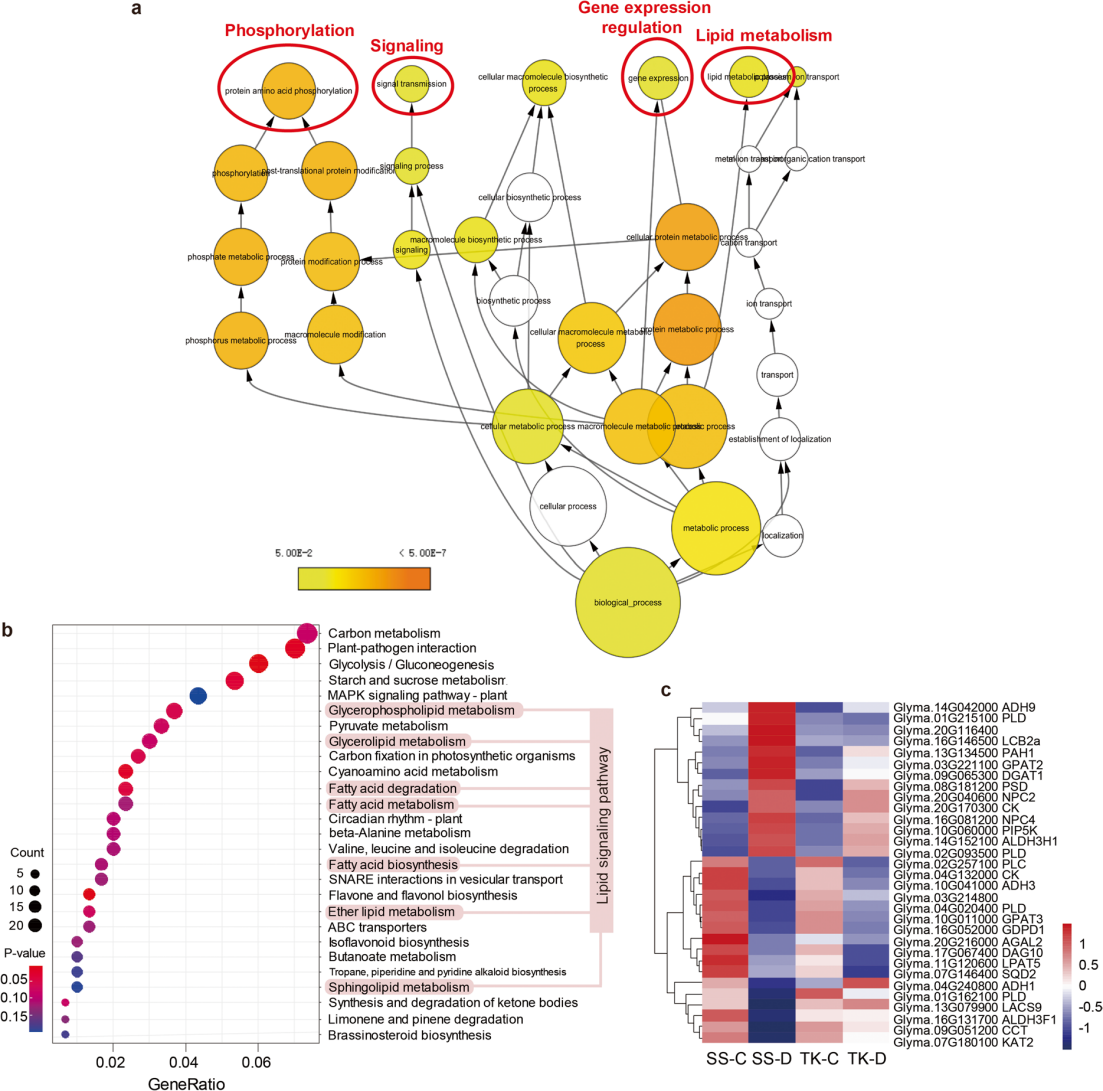
Gene ontology (GO) terms and Kyoto Encyclopedia of Genes and Genomes (KEGG) pathways associated with signaling and metabolism were enriched in SS2-2-specific DEGs. **a** Network pathway of gene ontology (GO) terms overrepresented among SS2-2-specific DEGs. **b** KEGG pathway enrichment analysis for SS2-2 specific DEGs. Dot size represents the gene number; dot color represents the *P* value. **c** Heatmap showing levels of expression of genes in KEGG lipid metabolism pathways overrepresented among DEGs

To understand the molecular mechanisms underlying the potential of SS2-2 in tolerating drought stress, we therefore investigated the gene functions and biological processes of the 2748 SS2-2-specific DEGs, using the Cytoscape plugin tool BiNGO to analyze overrepresented GO terms. The enriched GO categories were visualized in the form of a biological network (Fig. 3a). Several terms including “phosphorylation,” “signaling,” “gene expression regulation,” and “lipid metabolism” were significantly overrepresented when mapped against the BiNGO database. The SS2-2-specific DEG set was also subjected to KEGG pathway enrichment analysis. This identified 27 overrepresented pathways, several of which involved aspects of lipid metabolism, including “glycerophospholipid metabolism,” “glycerolipid metabolism,” “ether lipid metabolism,” and “sphingolipid metabolism” (Fig. 3b). Notably, genes involved in the GO term “signaling” also acted in pathways identified as enriched in the KEGG analysis, namely “MAPK signaling” and “phosphatidylinositol signaling” (Fig. S5). Thus, the results of both the GO and KEGG enrichment analyses indicated that pathways involved in lipid signaling and metabolism played critical roles in mediating the drought tolerance of SS2-2.

### Well-known abiotic stress-related DEGs in SS2-2

The 2748 SS2-2-specific DEGs included many well-known abiotic stress-related genes, including genes involved in signaling, reactive oxygen species (ROS) manipulation, and nucleotide-binding site and leucine-rich repeats (NBS-LRRs) (Table S4). Several genes involved in mitogen-activated protein kinase (MAPK) cascade in response to stress signals were identified among the SS2-2-specific DEGs, including two *MAPKKKs* (Glyma.03G237600 and Glyma.12G192500), one *MAPKK* (Glyma.15G172600), and three *MPKs* (Glyma.04G029700, Glyma.05G211000, and Glyma.16G032900). Other genes specifically upregulated in SS2-2 under drought stress encoded a plant hormone receptor, *ETHYLENE RESPONSE* (*ETR*; Glyma.09G002600), and its binding proteins, *ETHYLENE INSENSITIVE 3* (*EIN3*)-*BINDING F BOX PROTEIN 1* (*EBF*; Glyma.06G068400) and *BRASSINOSTEROID INSENSITIVE 1 (BRI1)-ASSOCIATED RECEPTOR KINASE* (*BAK*; Glyma.05G119500 and Glyma.15G051600), which trigger the phosphorylation cascade (Ouaked et al. 2003; Kim et al. 2012; Zhang et al. 2018). Other upregulated DEGs included six genes encoding key components in Ca^2+^ signaling, such as Calmodulins(-like) (Glyma.02G275600, Glyma.09G236800, Glyma.07G229500, and Glyma.14G040600) and cyclic nucleotide-gated channels (Glyma.19G255300 and Glyma.09G168700). Other DEGs that were more positively activated in SS2-2 than in Taekwang regulated ROS, an important messenger in abiotic stress responses. These genes included *RESPIRATORY BURST OXIDASE HOMOLOG B* (*RBOHB*; Glyma.03G236300), *ALDEHYDE DEHYDROGENASE* (*ALDH*; Glyma.14G152100), and *COPPER AMINE OXIDASE* (*CuAO*; Glyma.15G276400), which are essential for maintaining homeostasis in ROS levels, indicating that this mechanism was involved in drought stress responses in soybean (Qu et al. 2014; Raja et al. 2017; Raza et al. 2022). The genes *LIPOXYGENASE* (*LOX*; Glyma.10G153900 and Glyma.12G054700) and *HYDROPEROXIDE LYASE* (*HPL*; Glyma.12G191400), which act in linolenic acid metabolism pathways involved in ROS signaling, were differentially expressed in SS2-2. Lastly, 13 genes encoding *TOLL/INTERLEUKIN-1 RECEPTOR NBS-LRRs* (*TIR-NBS-LRRs*) and four genes encoding *COILED-COIL NBS-LRR*s (*CC-NBS-LRRs*) were differentially expressed during drought stress in SS2-2, relative to Taekwang (Table S4).

### Differential expression of TF genes in SS2-2

The GO term “gene expression regulation” was enriched among SS2-2-specific DEGs. These genes mostly encoded transcription factors (TFs) (Fig. 3a) belonging to seven soybean TF families, GmbZIP, GmDREB, GmERF, GmHDZIP, GmMYB, GmNAC, and GmWRKY (Fig. S6). Severe water deficiency was associated with strong upregulation of one *GmbZIP*, one *GmDREB*, one *GmHDZIP*, five *GmMYB*, six *GmNAC*, and twenty-six *GmWRKY* genes in leaf tissues of SS2-2 plants in the vegetative stage. In contrast, the expression of three *GmbZIP*s, one *GmDREB*, six *GmERF*s, five *GmHDZIP*s, five *GmMYB*s, seven *GmNAC*s, and two *GmWRKY*s was downregulated in SS2-2 under drought, but their expression showed no significant changes in Taekwang (Fig. S6). In addition to TFs that were specifically expressed in SS2-2, we also identified a set of TF genes that differentially expressed in both SS2-2 and Taekwang under drought stress (Table S5), implying some TFs showed a universal response to drought stress across soybean genotypes. Our results suggest, however, that a particular subset of TF gene families was involved in the regulation of the specific genes responsible for the high tolerance of drought stress found in SS2-2.

### The lipid-signaling pathway is involved in tolerance to drought in SS2-2

The GO and KEGG enrichment analyses identified a set of SS2-2-specific DEGs involved in lipid-signaling and metabolism pathways. This set included genes acting in the glycerolinositol signaling pathway, glycerolipid metabolism pathway, and glycerophospholipid metabolism pathway. These genes were investigated in detail (Fig. 3b; Table 1). We identified all the genes involved in lipid-signaling pathways whose expression differed significantly in SS2-2 under drought stress, compared to the control treatment, but showed no significant changes in Taekwang (Table 1). They included four genes encoding *PHOSPHOLIPASE D* (*PLD*s; Glyma.01G215100, Glyma.02G093500, Glyma.04G020400, and Glyma.01G162100), a *PHOSPHATIDYL INOSITOL MONOPHOSPHATE 5 KINASE* (*PIP5K*, Glyma.10G060000), two *NON-SPECIFIC PHOSPHOLIPASE C* genes (*NPC*; Glyma.16G081200, Glyma.20G040600), four phospholipid biosynthesis genes (Glyma.19G218100, Glyma.06G039200, Glyma.15G034100, and Glyma.08G181200), a sphingoid metabolism enzyme (Glyma.16G146500), *PHOSPHATIDYLINOSITOL PHOSPHOLIPASE C* (*PLC*; Glyma.02G257100), and DIACYLGLYCEROL KINASE (*DGK*; Glyma.06G254900). The heatmap of their expression is shown in Fig. 3c. Expression of these genes increased or decreased greatly in SS2-2 under drought stress while Taekwang showed no significant changes, indicating the importance of the lipid-signaling pathway in plant responses to abiotic stress in SS2-2.

**Table 1:** Differentially expressed genes involved in lipid metabolism pathways

Pathway name	Gene ID	Gene definition	log_2_(FoldChange)		FPKM				*A. thaliana* homolog
			SD/SC	SD/TD	SC	SD	TC	TD	
Glycerophospholipid metabolism	Glyma.03G221100	Glycerol-3-phosphate acyltransferase 2 (GPAT2)	3.00	1.28	0.08	0.44	0.07	0.17	AT1G02390.1
	Glyma.04G132000	Probable choline kinase 2 (CK)	−2.80	−0.67	0.21	0.02	0.13	0.03	AT1G74320.1
	Glyma.08G181200	Phosphatidylserine decarboxylase 2 (PSD)	1.36	0.65	14.80	41.40	11.16	27.11	AT5G57190.1
	Glyma.09G051200	Phosphorylcholine cytidylyltransferase (CCT)	−1.39	−1.11	18.85	7.73	18.95	15.42	AT2G32260.1
	Glyma.10G011000	Glycerol-3-phosphate acyltransferase 3 (GPAT3)	−5.30	−1.57	31.65	0.75	19.77	2.14	AT4G01950.1
	Glyma.11G120600	Lysophosphatidyl acyltransferase 5 (LPAT5)	−2.31	1.91	0.55	0.11	0.25	0.03	AT3G18850.1
	Glyma.13G134500	Phosphatidate phosphatase (PAH1)	1.14	0.54	1.78	4.08	1.62	2.61	AT5G42870.1
	Glyma.16G052000	Glycerophosphodiester phosphodiesterase (GDPD1)	−2.18	−0.52	1.30	0.29	1.07	0.41	AT5G41080.1
	Glyma.17G067400	Diacylglycerol kinase 10 (DGK10)	−1.40	0.15	24.69	9.38	17.00	7.95	AT4G30340.1
	Glyma.20G170300	Choline kinase (CK)	1.41	0.12	1.38	3.81	1.72	3.38	AT1G74320.1
Glycerolipid metabolism	Glyma.03G214800	Alpha/beta-Hydrolases superfamily protein	−2.03	−0.97	0.80	0.19	0.63	0.40	AT3G62860.1
	Glyma.07G146400	Sulfoquinovosyltransferase 2 (SQD2)	−3.24	−0.26	1.00	0.50	0.70	0.34	AT5G01220.1
	Glyma.09G065300	Diacylglycerol acyltransferase 1 (DGAT1)	1.33	0.72	0.91	2.39	1.10	1.37	AT2G19450.1
	Glyma.14G152100	Aldehyde dehydrogenase 3H1 (ALDH3H1)	1.51	0.36	14.80	45.96	16.78	34.36	AT1G44170.1
	Glyma.16G131700	Aldehyde dehydrogenase 3F1 (ALDH3F1)	−1.02	−0.64	19.27	10.01	15.17	14.82	AT4G36250.1
	Glyma.20G116400	Alpha/beta-Hydrolases superfamily protein	1.21	1.42	3.78	9.12	3.39	3.29	AT1G77420.1
Fatty acid degradation	Glyma.04G240800	Alcohol dehydrogenase 1 (ADH1)	−2.18	−2.69	0.96	0.22	0.49	1.35	AT1G77120.1
	Glyma.07G180100	Peroxisomal 3-ketoacyl-CoA thiolase 2 (KAT2)	−1.40	−0.93	22.48	9.04	20.04	16.35	AT2G33150.1
	Glyma.10G041000	Alcohol dehydrogenase 3 (ADH3)	−3.12	−1.13	0.49	0.05	0.30	0.12	AT5G43940.1
	Glyma.13G079900	Long chain acyl-CoA synthetase 9 (LACS9)	−1.98	−2.44	19.49	5.19	20.62	25.53	AT1G77590.1
	Glyma.14G042000	Alcohol dehydrogenase 9 (ADH9)	2.06	1.74	0.63	2.79	0.13	0.76	AT5G42250.1
Ether lipid metabolism	Glyma.01G215100	Phospholipase D gamma 1 (PLD)	1.60	2.64	2.46	7.62	1.38	1.18	AT2G42010.1
	Glyma.02G093500	Phospholipase D gamma 1 (PLD)	2.04	0.37	0.32	1.39	0.45	1.00	AT2G42010.1
	Glyma.04G020400	Phospholipase D delta (PLD)	−2.38	−0.88	0.53	0.10	0.41	0.18	AT4G35790.2
	Glyma.01G162100	Phospholipase D delta (PLD)	−1.03	−0.84	11.08	5.79	14.86	9.59	AT4G35790.2
	Glyma.20G040600	Non-specific phospholipase C2 (NPC2)	1.01	0.15	3.53	7.52	2.82	6.45	AT2G26870.1
	Glyma.16G081200	Non-specific phospholipase C4 (NPC4)	2.26	0.57	0.32	1.49	0.33	0.91	AT3G03530.1
	Glyma.02G257100	Phosphoinositide phospholipase C 6 (PLC)	−3.12	−0.51	0.20	0.02	0.21	0.02	AT2G40116.1
	Glyma.10G060000	Phosphatidylinositol 4-phosphate 5-kinase 9 (PIP5K)	1.27	0.33	8.29	21.13	8.82	15.69	AT3G09920.1
Sphingolipid metabolism	Glyma.20G216000	Alpha-galactosidase 2 (AGAL2)	−2.53	−1.54	0.22	0.04	0.09	0.05	AT5G08370.1
	Glyma.16G146500	Long chain base 2a (LCB2a)	1.05	1.06	8.06	17.86	8.37	8.20	AT5G23670.1

### RNA-seq validation by qRT-PCR analysis

**Fig. 4:**
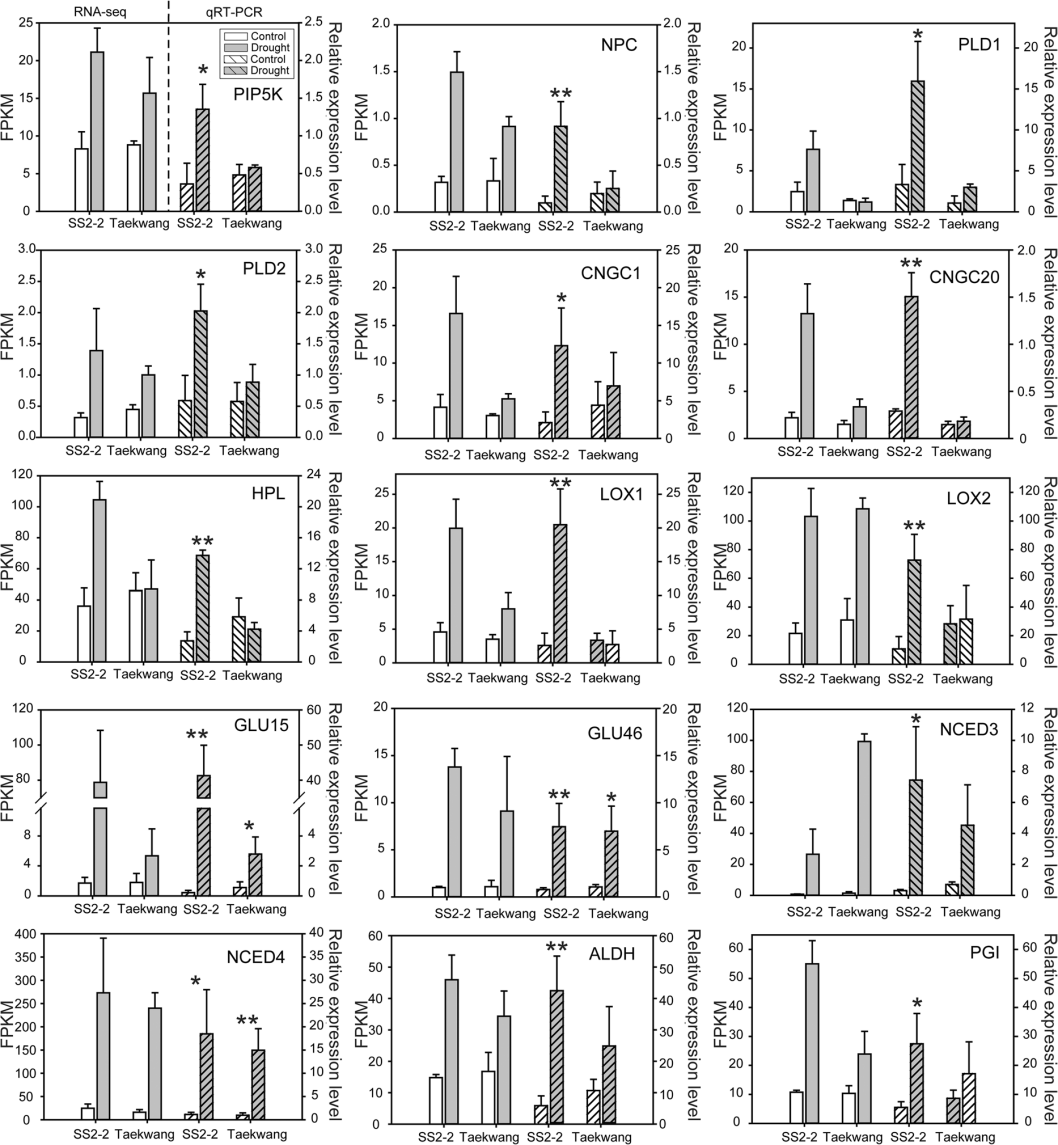
Comparison of RNA-seq data with qRT-PCR analysis. Each bar chart shows the enrichment of its transcript in the RNA-seq data, demonstrated by fragments per kilobase per million mapped reads (FPKM) value on the left Y-axis and the fold change of the gene relative expression level obtained by qRT-PCR on the right Y-axis. *Significant at *P* < 0.05; **significant at *P* < 0.01

The RNA-seq data were confirmed by qRT-PCR analysis of a subset of 15 genes selected from the SS2-2-specific DEGs. The selected subset included four lipid metabolism pathway genes (*PIP5K*, Glyma.10G060000; *NPC*, Glyma.16G081200; and two *PLDβ1*s, Glyma.01G215100 and Glyma.02G093500), two calcium channel genes (*CNGC1*, Glyma.19G255300; *CNGC20*, Glyma.09G168700), three alpha-linolenic acid metabolism pathway genes (*LOX1*, Glyma.10G153900; *LOX2*, Glyma.12G054700; and *HPL*, Glyma.12G191400), and *ALDEHYDE DEHYDROGENASE* (*ALDH*, Glyma.14G152100). The qRT-PCR analysis confirmed that all these genes were significantly upregulated in SS2-2 in response to drought stress (Fig. 4). In addition, a dot plot of the fold change in expression of 15 genes revealed a high level of consistency between the relative expression levels obtained from the RNA-seq and qRT-PCR analyses. A regression analysis of these data revealed a correlation coefficient of 0.6633 (Fig. S7).

### Functional validation of drought stress resistance genes in *Arabidopsis* mutants

To confirm the function of DEGs identified as involved in lipid signaling, we investigated drought tolerance in three *Arabidopsis* knockout mutants, *pip5k* (AT3G09920), *pldβ*1 (AT2G42010), and *npc* (AT3G03530), which are the homologs of *GmPIP5K* (Glyma.10G060000), *GmPLDβ1* (Glyma.01G215100), and *GmNPC* (Glyma.16G081200), respectively. The germination rate of *pip5k* mutants was significantly lower than that of wild-type seeds on medium containing 300 mM mannitol, which mimics drought stress, but germination rates of *pld* and *npc* mutants did not differ significantly from wild-type seeds (Fig. S8). This suggests that only *PIP5K* functioned in response to drought stress.

**Fig. 5:**
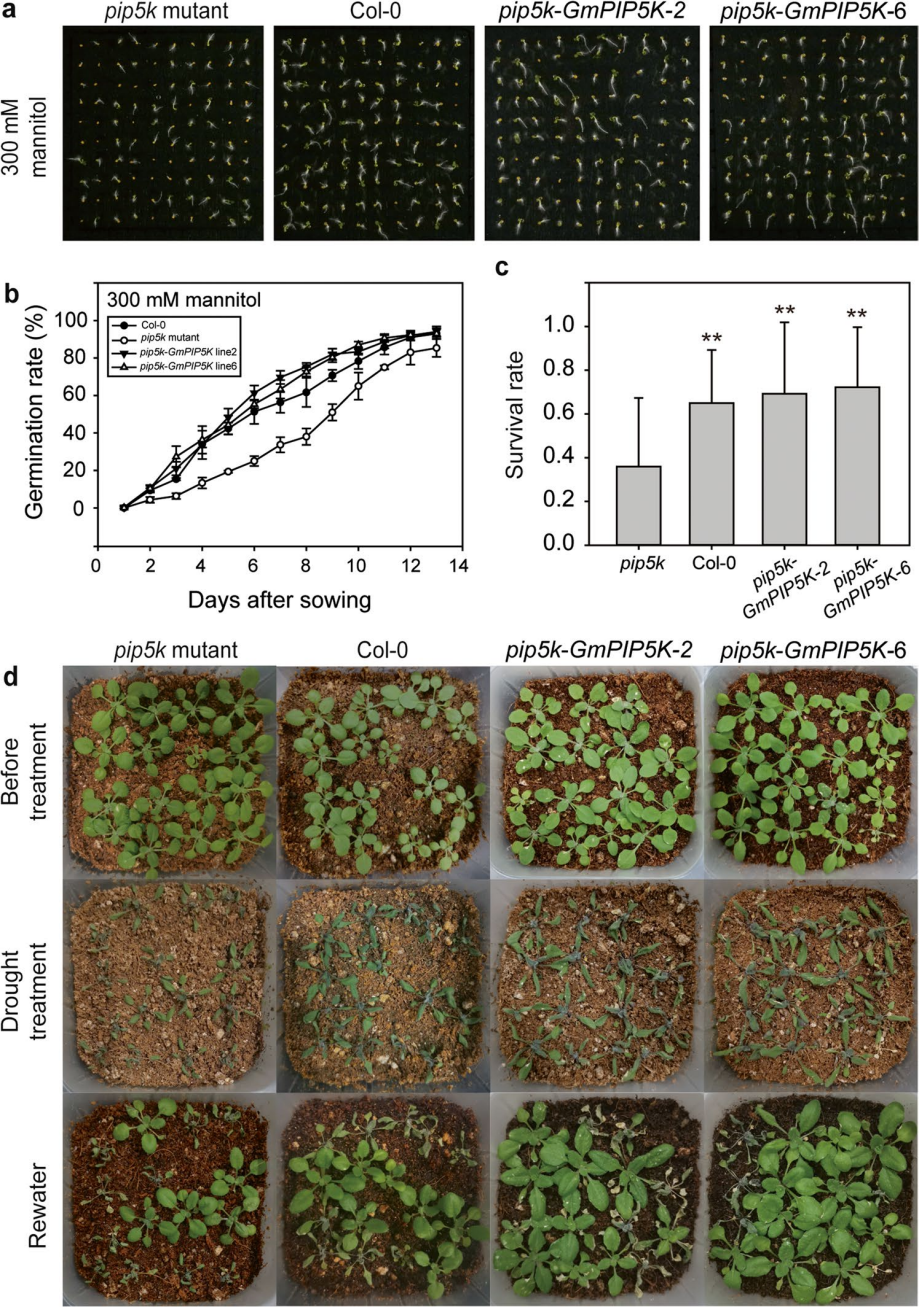
Restoration of *GmPIP5K* function conferred drought tolerance in *Arabidopsis* mutants**. a** Representative photographs of Col-0, *pip5k*, *pip5k-GmPIP5K* line 2, and *pip5k-GmPIP5K* line 6 *Arabidopsis* seeds grown for 14 days on 0.5× MS medium containing 300 mM mannitol to induce drought stress. **b** Germination rates of the Col-0, *pip5k, pip5k-GmPIP5K* line 2, and *pip5k-GmPIP5K* line 6 seeds on medium containing 300 mM mannitol. **c** Seedling survival rates of the Col-0, *pip5k*, *pip5k-GmPIP5K* line 2, and *pip5k-GmPIP5K* line 6 after drought treatment and rewatering. *Significant at *P* < 0.05. **d** Representative photographs of the Col-0, *pip5k*, *pip5k-GmPIP5K* line 2, and *pip5k-GmPIP5K* line 6 seedlings before drought treatment and after 10 days of drought treatment and rewatering

To confirm the function of *GmPIP5K*, we transformed *Arabidopsis pip5k* mutants with the *GmPIP5K* coding sequence promoted by CaMV35S promoter to generate transgenic plants constitutively expressing *GmPIP5K*. We selected two lines of T_3_ plants, designated *pip5k*-*GmPIP5K*-2 and *pip5k*-*GmPIP5K*-6, for further analysis and examined germination and seedling survival rates of Col-0, *pip5k*, *pip5k*-*GmPIP5K*-2, and *pip5k*-*GmPIP5K*-6 plants under drought stress. The decreased germination rate of *pip5k* mutants growing on 300 mM mannitol medium was rescued in transgenic lines expressing *GmPIP5K* (Fig. 5a, b). In addition, we exposed seedlings to drought stress for 10 days, before rewatering and measuring survival rates. The *pip5k* mutants showed significantly lower survival rates (34.9%) than Col-0 (69.2%) or *pip5k*-*GmPIP5K*-2 (66.1%) and *pip5k*-*GmPIP5K* -6 (72.2%) after rewetting (Fig. 5c, d). This indicated that *GmPIP5K* may play a major role in enhancing tolerance to abiotic stress.

## Discussion

This study was designed to identify genes involved in drought tolerance in soybean. We initially measured drought tolerance in 11 different soybean cultivars and identified SS2-2 and Taekwang as the most drought-tolerant and drought-sensitive genotypes, respectively. These soybean genotypes have been used as the parental lines for a RIL population for quantitative trait locus analysis, which should facilitate future genetic mapping of genes controlling drought tolerance. We conducted a transcriptomic analysis of SS2-2 and Taekwang and identified, with high confidence, 2748 DEGs that were specifically upregulated in SS2-2 in response to severe water restriction during the vegetative stage (Fig. 2, Table S3).

Several transcriptomic data with drought stress treatment have been released in soybean. To investigate whether the SS2-2-specific DEGs identified in this study also respond to drought stress in other cultivars, we examined the expression levels of our DEGs that are involved in lipid metabolism in other transcriptomic data, including drought-tolerant cultivars Jindou 21 and LX, drought-sensitive cultivars Tianlong No.1 and Williams 82, and cultivars Heike68 and Magellan with no reported resistance to stress (Fig. S9). We found that a large number of genes had significant differential expression between control and stress conditions in drought-tolerant cultivars than in drought-sensitive cultivars. Notably, Glyma*.04G240800 (ADH)* and *Glyma.10G01100 (GPAT3)* showed differential expression in several genotypes, suggesting that these two genes may have universal function in drought stress response. Interestingly, Heike68, an early-maturing elite cultivar had the most genes overlapping with our lipid metabolism-related DEGs, but no study has reported that it is resistant to stress. Therefore, further investigation is needed to determine whether Heike68 has similar phenotypes and responses to SS2-2 under drought stress.

Seven different TF families were included in SS2-2 specific DEGs with the WRKY family having the greatest number. Several dozens of the ~180 *WRKY* genes identified in the soybean reference genome are known to respond to dehydration and salinity in the Williams 82 cultivar (Song et al. 2016a; Yu et al. 2016), and this set of genes overlapped with the WRKY TFs induced by drought stress in both SS2-2 and Taekwang (Table S5). The set of SS2-2-specific WRKY TFs, however, differed from such general drought-responsive WRKYs (Table S5, Fig. S6) and were thus more likely to play important roles in drought tolerance signaling cascades. No previous comparative transcriptome study of two phenotypically different soybean cultivars analyzed during their vegetative or reproductive stages has indicated such a significant role of WRKY TFs in drought tolerance (Chen et al. 2013; Le et al. 2011; Prince et al. 2015). Some *WRKY* genes, such as *OsWRKY30* in rice, are activated by MAPK cascades when plants are exposed to drought stress (Shen et al. 2012), although phosphorylation of WRKY proteins by MAPKs under abiotic stress conditions has been reported less frequently than in the response to biotic stress (Banerjee and Roychoudhury 2015). We found upregulation of *GmWRKY30* (Glyma.17G222300) in SS2-2 under drought stress but downregulation of this gene in Taekwang (Table S5).

WRKY proteins act, directly or indirectly, to regulate the expression of a series of genes involved in ABA signaling and ROS scavenging, thus leading to drought tolerance (Li et al. 2020; Viana et al. 2018). The binding of *AtWRKY57* to the W-box element in the promoters of *RESPONSIVE TO DESICCATION 29* (*RD29*) and *9-CIS-EPOXYCATOTENOID DIOXYGENASE 3* (*NCED3*) increases their expression, resulting in elevated ABA levels and conferring drought tolerance in *Arabidopsis* (Jiang et al. 2012). Studies of transgenic plants overexpressing *ZmWRKY58* or *GsWRKY20* suggest these genes act as positive regulators mediating responses to drought of maize (*Zea mays* L*.*) and wild soybean (*Glycine soja*), respectively (Cai et al. 2014; Ning et al. 2017). The soybean homologs of these two genes (*GmWRKY58* and *GmWRKY20*) showed specific upregulation in SS2-2 during drought (Fig. S6). In contrast, homologs of other SS2-2 specific WRKY TFs function as negative regulators, including *GhWRKY33* in cotton (*Gossypium hirsutum*) (Wang et al. 2019), *AtWRKY60* in *Arabidopsis* (Chen et al. 2010), and *SlWRKY81* in tomato (*Solanum lycopersicum*) (Ahammed et al. 2020). These contrasting results indicate that careful functional validation of different SS2-2-specific WRKY TFs is required if a precise understanding of their involvement in the signaling pathways controlling drought tolerance of soybean is to be obtained.

Receptors in cell membranes detect signals, allowing plants to respond to environmental stimuli such as drought stress. Lipids are major components of the membrane and act as a rich pool for generating signaling messengers, including phosphatidic acid, phosphoinositide, sphingolipids, and free fatty acids, via lipid metabolism pathways (Lim et al. 2017). Lipids are combined with diverse hydrophobic and hydrophilic elements and are classified according to these structures into groups such as fatty acids, glycerolipids, sphingolipids, sterol lipids, phenol lipids, saccharolipids, and polyketides (Hou et al. 2016). Fatty acid metabolism mediates signal transduction in plant defense (Lim et al. 2017), and fatty acid degradation and biosynthesis pathways were enriched among our DEGs (Fig. 3b), probably because of crosstalk between the biotic defense and abiotic stress responses. The KEGG analysis of our DEG data identified several genes involved in the biosynthesis or degradation of sphingolipids and fatty acids, including *AGAL2*, *LCB2a*, *ADHs*, *KAT*, and *LACS* (Table 1). As for phosphorus-containing glycerolipids which include phosphatidylinositol (PI), phosphatidylcholine (PC), phosphatidylethanolamine (PE), phosphatidylglycerol (PG), and phosphatidylserine (PS), they mainly serve as structural components of biological membranes. Besides functioning as structural lipids, PI and PC can generate a cellular signal molecule, phosphatidic acid (PA), in plants facing abiotic stress (Yao and Xue 2018). Many of our DEGs were involved in glycerolipid metabolism and acted in glycerolipid biosynthesis, remodeling, or degradation pathways. For example, several acyltransferases, including GPAT2, GPAT3, and LPAT5, are reported to be responsible for glycerophospholipid biosynthesis in *Arabidopsis* (D’Auria 2006); CCT and CKs regulate levels of PC, a glycerophospholipid substrate (Craddock et al. 2015; Lin et al. 2019); GDPD and several alpha/beta-hydrolases are responsible for glycerophospholipid degradation; DGK10, PAH1, and DGAT1 are involved in diacylglycerol metabolism, which is involved in signaling under stressful conditions (Yoshitake et al. 2017; Tan et al. 2018) (Table 1). These DEGs may function in responding to stress through regulating lipid signaling pathways. Noticeably, the phospholipase D signaling pathway, which hydrolyzes PI to produce a lipid secondary messenger, showed the highest level of enrichment among our DEGs; in total, we identified four *PLDs*, two *NPCs*, one *PLC*, and one *PIP5K* that acted in this pathway (Table 1). Through further genetics study, we have discovered that overexpression of *GmPIP5K* enhances drought stress tolerance in *Arabidopsis*. It has been reported that plants contain high levels of *PI(4)P* and low levels of *PI(4,5)P*_*2*_ under normal conditions. However, under abiotic stress such as drought, levels of *PI(4,5)P*_*2*_ increase rapidly (Munnik and Testerink 2009). Therefore, we hypothesize that *GmPIP5K* responds to drought stress tolerance by generating *PI(4,5)P*_*2*_ in soybean. To confirm this hypothesis, measuring the levels of *PI(4)P* and *PI(4,5)P2* in *GmPIP5K* ectopic expression plants and wild-type plants is needed. Besides, although our study has confirmed the function of *GmPIP5K* by recovering it in the *Arabidopsis* mutant lines, it would be more convincing to overexpress it in *Arabidopsis* or even soybean to determine whether drought tolerance is enhanced. Furthermore, we believe that other DEGs related to lipid metabolism are also worth investigating with transgenic methods to understand their function in enhancing drought tolerance.

The qRT-PCR analysis showed significant upregulation of *GmPIP5K* during drought stress only in the cultivar SS2-2 (Fig. 4). An analysis of the *Arabidopsis pip5k* mutant confirmed that *GmPIP5K* functioned in responses to abiotic stress (Fig. 5)*.* Comparison of *GmPIP5K* sequences from the six cultivars used in the first screen provided insight into the different *GmPIP5K* expression patterns observed in these genotypes. Of the six cultivars, SS2-2, Williams 82, and Buseok were drought-tolerant, while Taekwang, Cheongja3, and Keonol were drought-sensitive (Fig. S2). Although the sequence of the coding region of *GmPIP5K* was identical in all six genotypes, 12 single nucleotide polymorphisms (SNPs) were present in the downstream sequence (420 to 1655 bp) of 3′ UTR, separating the cultivars into two haplotypes that exactly matched their drought tolerance and sensitivity phenotypes (Fig. S10). This indicated that *cis*-acting regulatory elements that regulate the expression of *GmPIP5K* were present in the downstream region of the gene, and the distinctive sequence differences between cultivars resulted in differential gene expression under drought stress, causing the observed differences in drought sensitivity or tolerance phenotypes. *Cis*-acting regulatory elements are noncoding regions of DNA that regulate the transcription of neighboring genes. Such elements include promoters, enhancers, and silencers. Promoters are always located upstream of the coding region, but enhancers may be located upstream or downstream of their target genes (Raatz et al. 2011; Weber et al. 2016). Other enhancers may be located downstream of the *GmPIP5K* coding region, but further studies, for instance, electrophoresis mobility shift assays, are required to confirm whether this region can be bound by activators such as TFs.

## Conclusion

We performed transcriptional profiling of two soybean cultivars that showed contrasting responses to drought stress. The transcriptomic data provided comprehensive information about the genes involved in regulating tolerance to drought stress during the vegetative stage in soybean, and identified a set of genes that were differentially expressed in the drought-tolerant cultivar SS2-2 under drought stress but whose expression did not change in Taekwang, a drought-sensitive cultivar. Lipid metabolism is involved in signal transduction, and, in particular, the generation of PA from phosphatidylinositol plays a central role in plant responses to abiotic stress. Analysis of *Arabidopsis* mutants confirmed that restoring *GmPIP5K* function promoted drought stress tolerance*.* Moreover, a set of TFs with differential expression specific to SS2-2 included many *WRKY* genes, whose role in conferring tolerance to abiotic stress remains to be explored. The DEGs identified in this study provide a major insight into the complicated drought tolerance mechanisms of plants, as well as provide promising genetic resources for soybean breeding programs to improve drought tolerance.

## Supplementary information

ESM 1 (XLSX 462 kb)

ESM 2 (DOCX 5489 kb)

## Data Availability

The datasets generated during and/or analyzed during the current study are available in the Short Read Archive under BioProject accession: PRJNA933767
