# Supplementary material for: RNA-seq profiling in leaf tissues of two soybean (Glycine max [L.] Merr.) cultivars that show contrasting responses to drought stress during early developmental stages
Source: Mol Breed. 2023 May 9;43(5):42. doi: 10.1007/s11032-023-01385-1 (PMC10248644; doi:10.1007/s11032-023-01385-1)
Supplement: Supplementary file 2 — (DOCX 5489 kb) [file 11032_2023_1385_MOESM2_ESM.docx]

*Journal name: Molecular Breeding*

RNA-seq profiling in leaf tissues of two soybean (*Glycine max* [L.] Merr.) cultivars that show contrasting responses to drought stress during early developmental stages

Xuefei Yang^a^, Hakyung Kown^b^, Moon Young Kim^b,c^, Suk-Ha Lee^b,c,*^

*^a^Key Laboratory of Herbage & Endemic Crop Biotechnology, Ministry of Education, School of Life Sciences, Inner Mongolia University, Hohhot 010000, China*

*^b^Department of Agriculture, Forestry and Bioresources and Research Institute of Agriculture and Life Sciences, Seoul National University, Seoul 08826, Republic of Korea*

*^c^Plant Genomics and Breeding Institute, Seoul National University, Seoul 08826, Republic of Korea*

*Corresponding author: Suk-Ha Lee

Address: Crop Genomics Lab. Rm. 4105 Bldg. 200 CALS, Seoul National University, 1 Gwanak-ro, Gwanak-gu, Seoul 08826, Republic of Korea.

Tel: +8228804545; Fax: +8228774550

E-mail address: yangxuefei1229@gmail.com (X. Yang), khg940711@snu.ac.kr (H. Kwon), moonykim@snu.ac.kr (M. Y. Kim), sukhalee@snu.ac.kr (S-H. Lee)


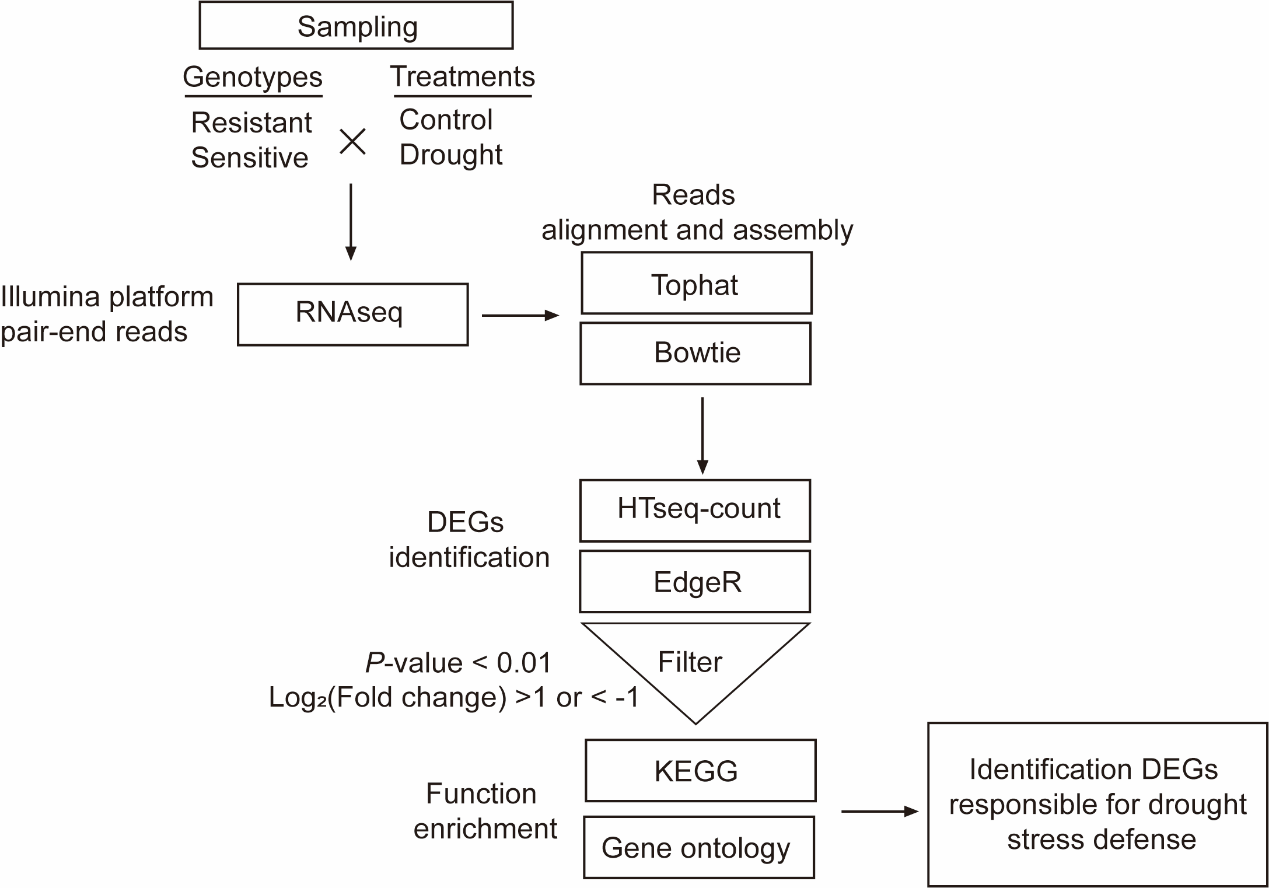


**Fig. S1 Workflow and experiment design**

Leaf samples were collected from two genotypes under two conditions. The cDNA libraries were sequenced using the Illumina platform with paired-end reads. The reads were indexed using Bowtie2 and mapped to the soybean reference genome using TopHat. The count number was calculated using the script HTseq-count in the HTseq package. The EdgeR program was used to test for statistically significant differences in transcript expression in four pairs of comparisons, within and between cultivars under control conditions and drought treatments. Differentially expressed genes (DEGs) were identified using the criterion of a twofold expression change (*P* < 0.001). The DEGs were analyzed using KEGG and GO enrichment analysis to identify candidate genes likely to be responsible for drought stress tolerance.


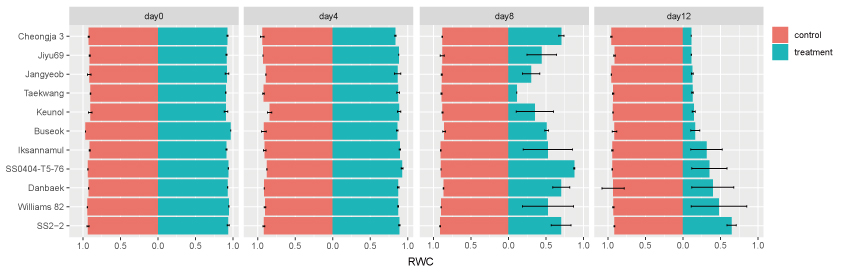


**Fig. S2 Consecutive measurement of the reduction in leaf relative water content in soybean cultivars subjected to drought stress**

Plants of 11 soybean cultivars (SS2-2, Williams 82, Danbaek, SS0404-T5-76, Iksannamul, Buseok, Keunol, Taekwang, Jangyeob, Jiyu69, and Cheongja 3) were grown until the V3 stage, at which point the water supply was restricted for 12 d. Second trifoliate leaves were collected and relative water content (RWC) was determined every 4 d. All well-watered (control) plants maintained a leaf RWC > 0.83 throughout the experiment. Water-restricted plants showed considerable depletion in leaf RWC beyond 4 d of water restriction, although the extent differed greatly between cultivars. Note that the RWC of Taekwang sharply dropped between days 4 and 8, with the mean RWC value falling from 0.87 to 0.11, while SS2-2 maintained the highest leaf RWC with a value of 0.65 on day 12, although, on day 8, RWC was highest in SS0404-T5-76.


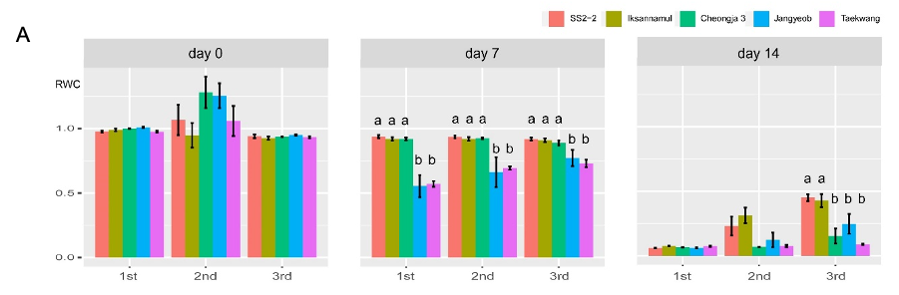


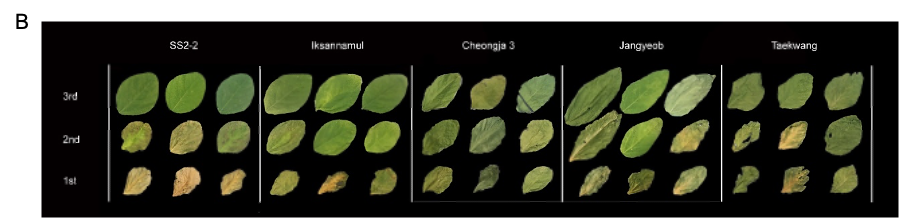


**Fig. S3 Second round screen of relative water content in trifoliate leaves of five soybean cultivars during drought stress**

(**A**). Changes in RWC in first, second, and third trifoliate leaves of five cultivars measured after 0, 7, or 14 days of water restriction. Duncan tests were followed by ANOVA to classify cultivars as resistant/tolerant or susceptible. One-way ANOVA was conducted by each day and trifoliate (alpha = 0.01) to determine significant differences in RWC between cultivars. On day 0, all trifoliate leaves were nearly saturated (RWC values > 0.93). Consistent with the results of the previous screen, leaves from Jangyeob and Taekwang wilted faster than those of other cultivars, and RWC in Cheongja 3 dropped sharply between days 7 and 14. Upper leaves had higher RWC values, indicating that soybean leaves dry from the bottom of the plant under water restrictive conditions. (**B**). The appearance of rewetted leaves from five soybean cultivars subjected to 14 d of drought stress. The leaves were collected and allowed to soak in water for 4 h. Only the third trifoliate leaves from SS2-2 and Iksannamul demonstrated a normal leaf shape after rewetting. Other severely dried leaves did not recover their tugor.


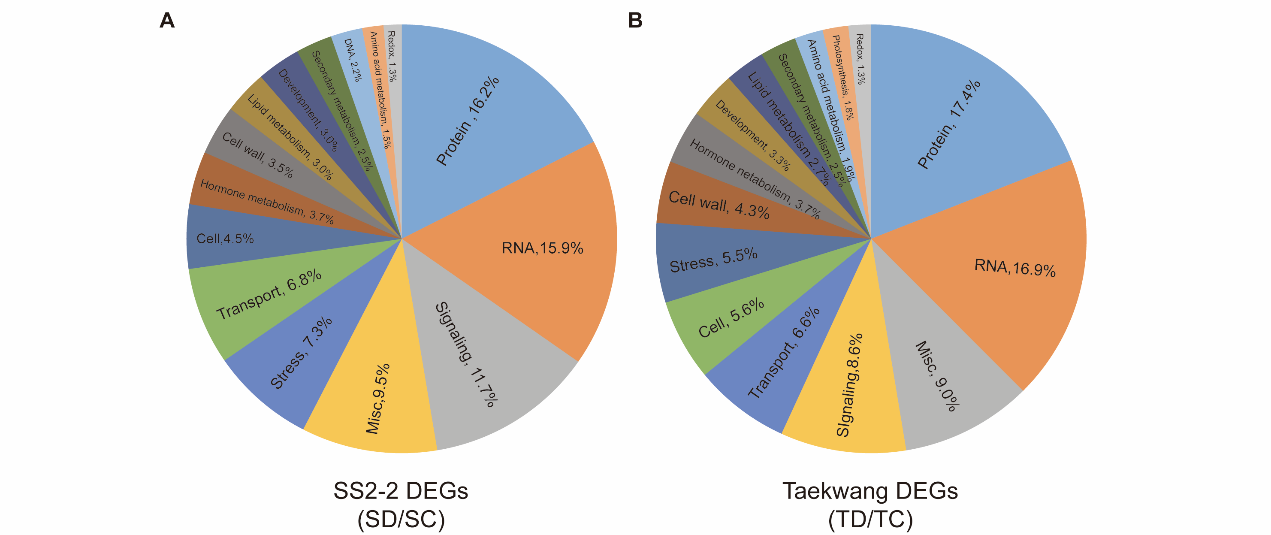


**Fig. S4 GoMapMan assignment of DEGs to functional categories**

The pie charts show the functional categorization of DEGs in SS2-2 and Taekwang assigned by GoMapMan. (**A**) DEGs identified in SS2-2. (**B**) DEGs identified in Taekwang. SC: SS2-2 under control; SD: SS2-2 under drought; TC: Taekwang under control; TD: Taekwang under drought.


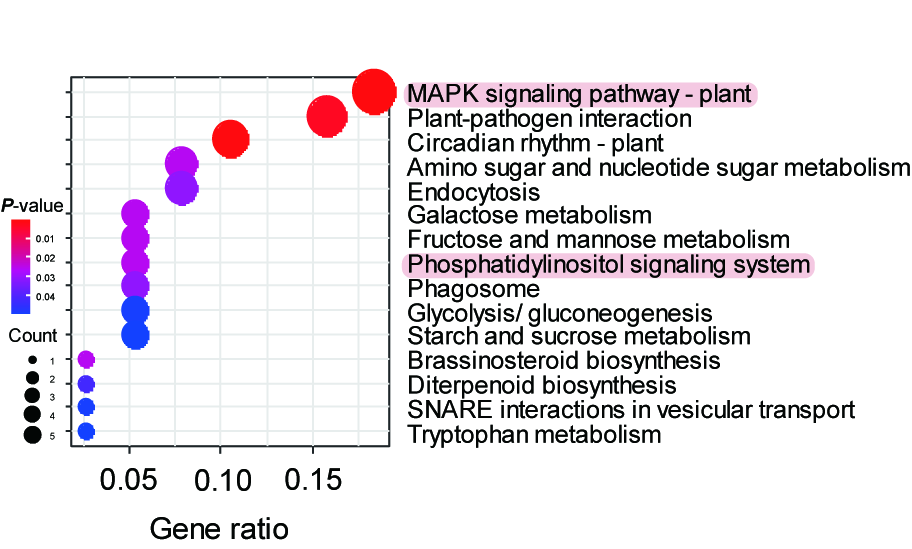


**Fig. S5 Kyoto Encyclopedia of Genes and Genomes (KEGG) pathway of genes associated with the term “signaling” in a gene ontology enrichment analysis**

The KEGG pathway enrichment analysis for the genes associated with the gene ontology term “signaling” in a GO analysis. The dot size represents the number of genes; the dot color represents the *P*-value.


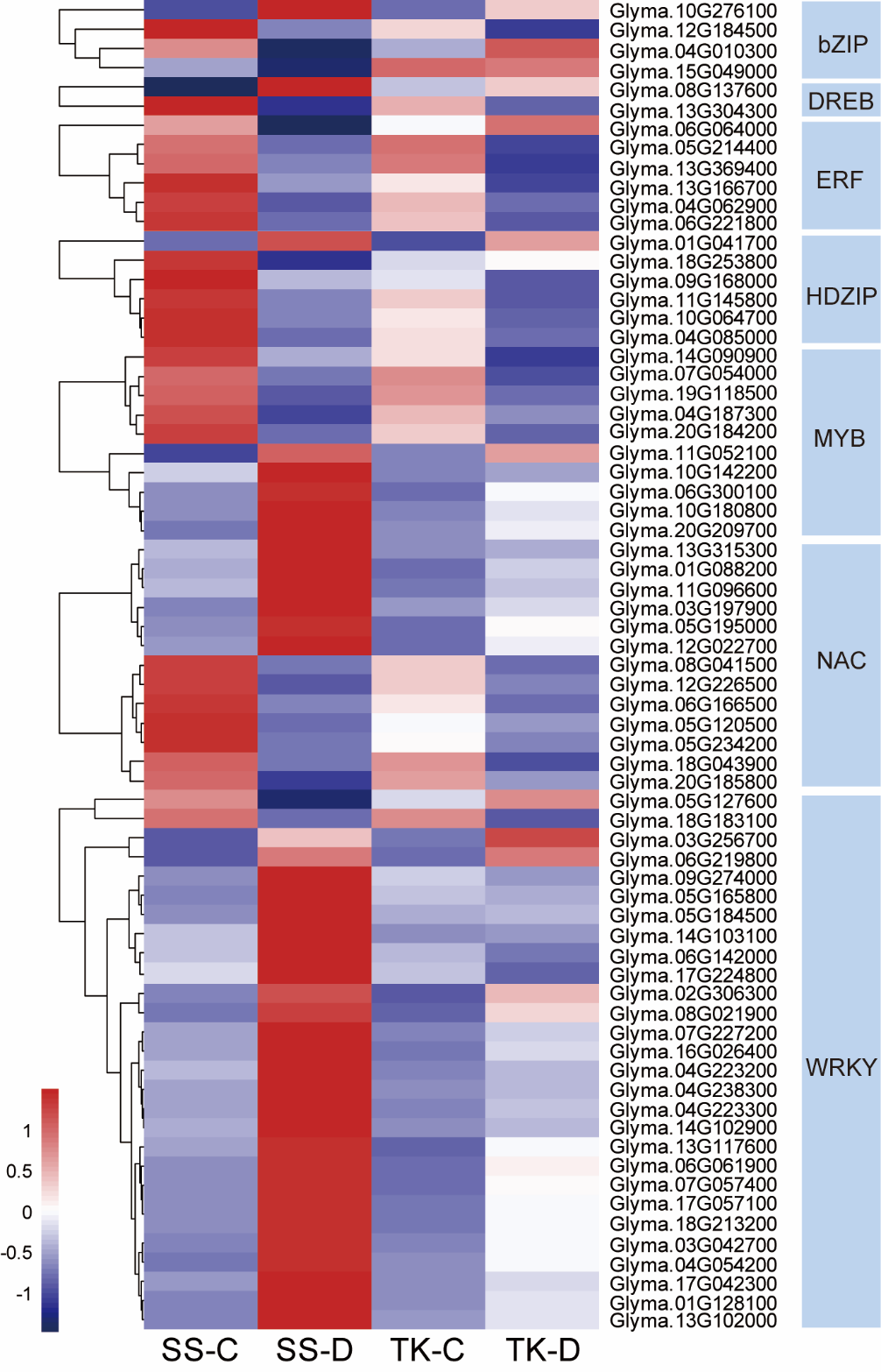


**Fig. S6 Transcription factors were enriched among SS2-2-specific DEGS** Heat map showing expression level changes of members of seven different soybean transcription factor families identified as SS2-2-specific DEGs.


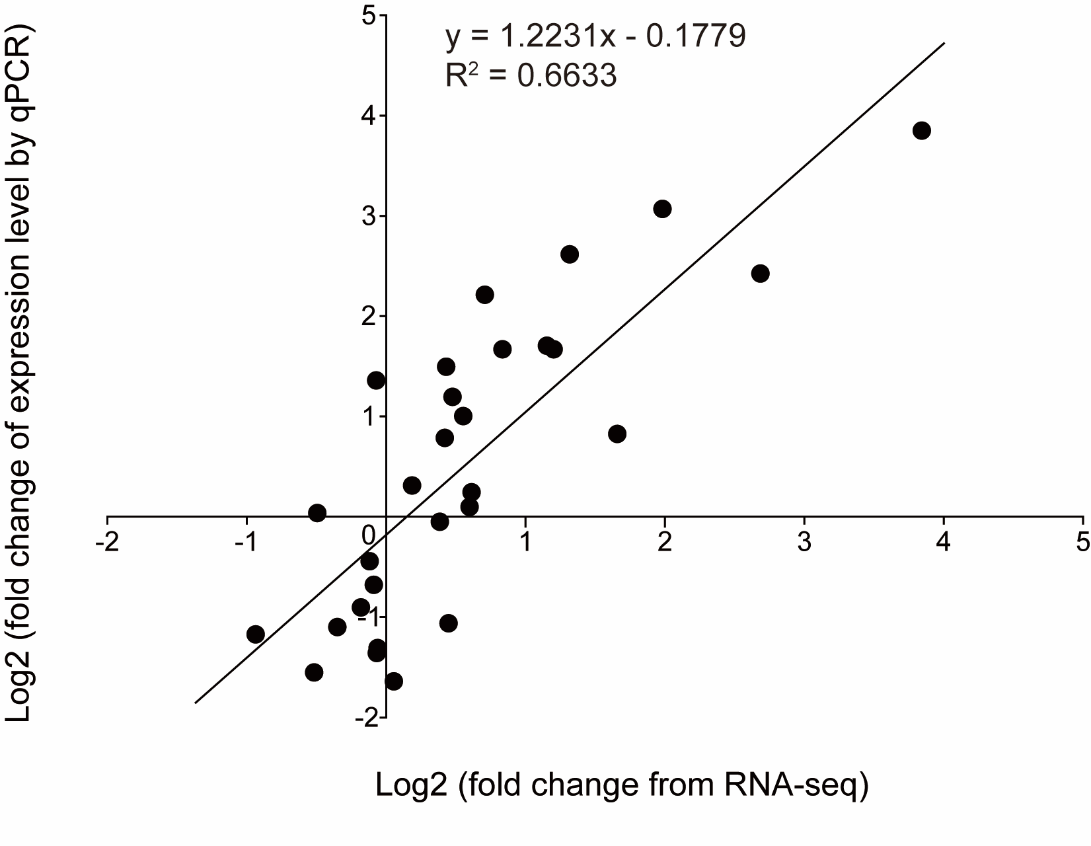


**Fig. S7** **Dot plot and linear regression analysis of qRT-PCR and transcriptomic data**

Dots represent log2-transformed fold change values of individual genes obtained from qRT-PCR analysis (Y-axis) and RNA-seq data (X-axis). The fold change (FC) was calculated as the ratio between the sample under drought stress and control. R^2^: correlation coefficient.


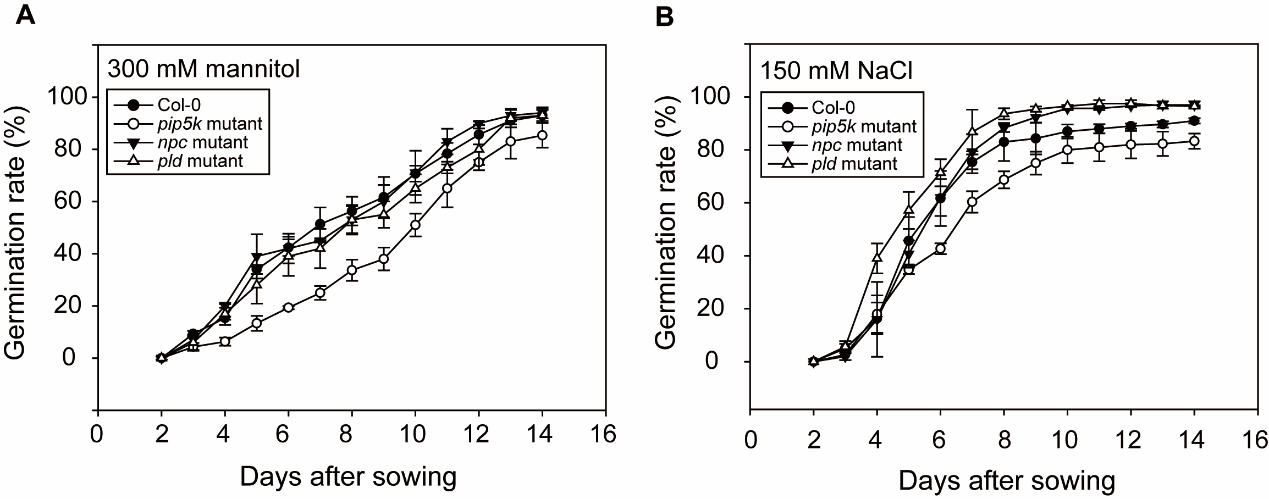


**Fig. S8 Germination rates of Col-0 and mutant Arabidopsis seeds under osmotic and salt stress**

(**A**) Seed germination rates of Col-0 (WT), *pip5k*, *npc,* and *pld* mutants 14 d after sowing on 300 mM mannitol medium. (**B**) Seed germination rates of Col-0, *pip5k*, *npc,* and *pld* mutants 14 d after sowing on 150 mM NaCl medium.


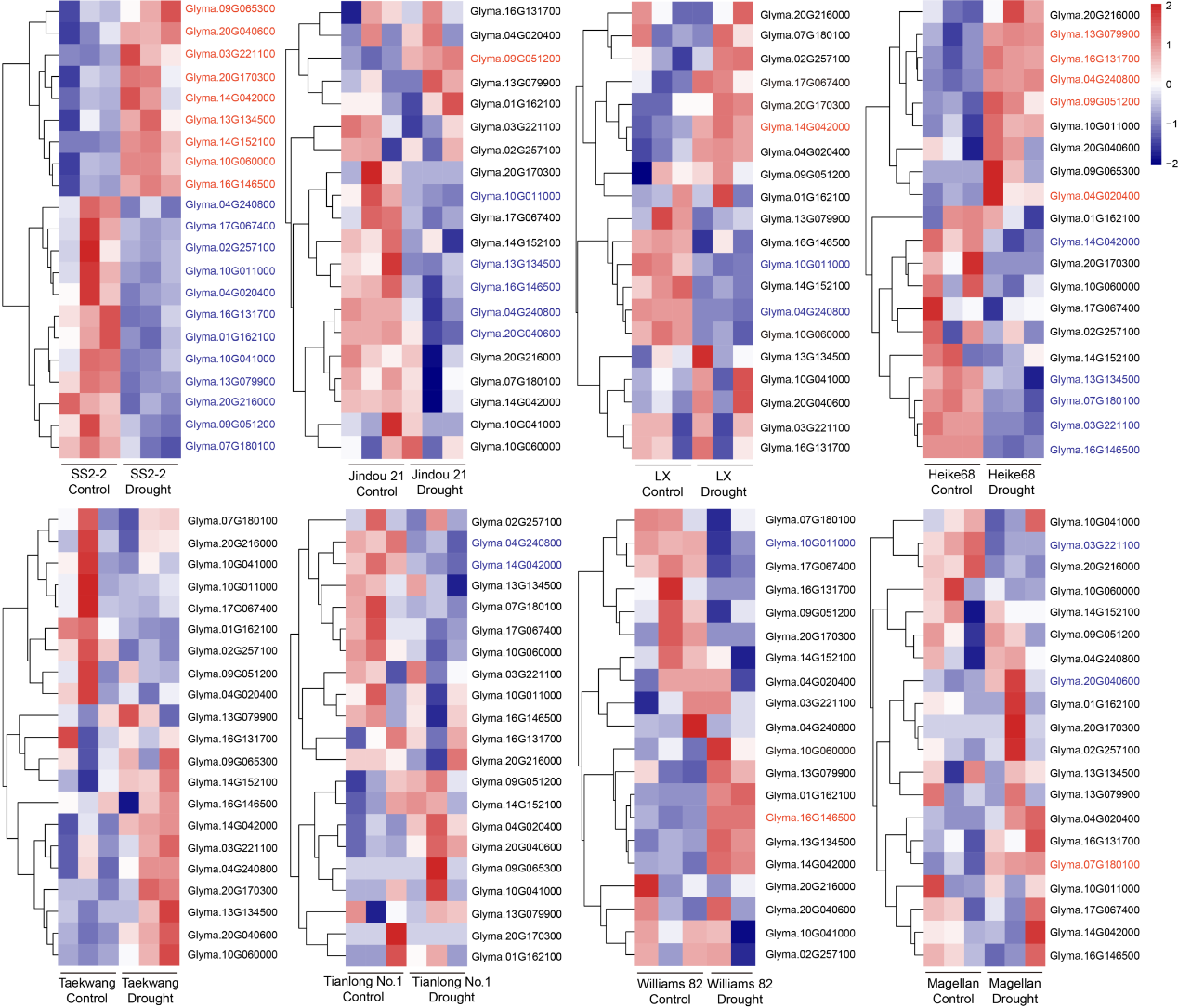
**Fig. S9 Heatmap illustrating expression level of lipid metabolism-related DEGs in other soybean cultivars**

The expression patterns of lipid metabolism-related DEGs in different soybean cultivars between control and drought stress were illustrated by heatmap. Eight cultivars included SS2-2, Jindou 21, LX (drought-tolerant cultivars), Taekwang Tianlong No.1 and Williams 82 (drought-sensitive cultivars), Heike68, and Magellan (resistance not mentioned). Genes that were up-regulated or down-regulated under drought stress were colored in red or blue, respectively.


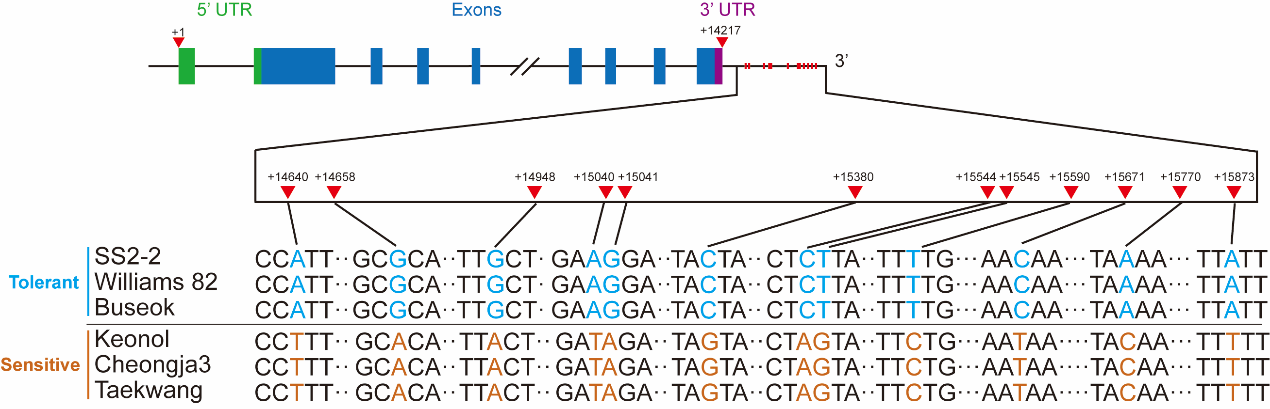


**Fig. S10 Sequence variation in *GmPIP5K* in drought-tolerant and drought-sensitive soybean cultivars**

Upper panel: map of the *GmPIP5K* locus; lower panel: sequences of 3ʹ downstream region of *GmPIP5K* obtained from six different soybean cultivars. Red arrows indicate the positions of single nucleotide polymorphisms (SNPs). Blue: drought-tolerant cultivars; brown: drought-sensitive cultivars.
